# Increased Antioxidant Capacity and Pro-Homeostatic Lipid Mediators in Ocular Hypertension—A Human Experimental Model

**DOI:** 10.3390/jcm9092979

**Published:** 2020-09-15

**Authors:** Mia Langbøl, Sarkis Saruhanian, Thisayini Baskaran, Daniel Tiedemann, Zaynab A. Mouhammad, Anne Katrine Toft-Kehler, Bokkyoo Jun, Rupali Vohra, Nicolas G. Bazan, Miriam Kolko

**Affiliations:** 1 Department of Drug Design and Pharmacology, University of Copenhagen, 2100 Copenhagen, Denmark; svh416@alumni.ku.dk (S.S.); gfk783@alumni.ku.dk (T.B.); wjf833@alumni.ku.dk (D.T.); zaynab.mouhammad@gmail.com (Z.A.M.); toft-kehler@dadlnet.dk (A.K.T.-K.); rvohra@sund.ku.dk (R.V.); 2 Neuroscience Center of Excellence, Louisiana State University Health New Orleans, New Orleans, LA 70112, USA; bjun@lsuhsc.edu (B.J.); nbazan@lsuhsc.edu (N.G.B.); 3 Department of Veterinary and Animal Sciences, University of Copenhagen, 2000 Frederiksberg, Denmark; 4 Department of Ophthalmology, Copenhagen University Hospital, Rigshospitalet-Glostrup, 2600 Glostrup, Denmark

**Keywords:** antioxidant capacity, oxidative stress, normal-tension glaucoma, ocular hypertension, hypoxia, lipidomics, pro-homeostatic lipid mediators, hydroxyeicosatetraenoic acids, hydroxydocosahexaenoic acids

## Abstract

The main risk factor for primary open-angle glaucoma (POAG) is increased intraocular pressure (IOP). It is of interest that about half of the patients have an IOP within the normal range (normal-tension glaucoma, NTG). Additionally, there is a group of patients with a high IOP but no glaucomatous neurodegeneration (ocular hypertension, OHT). Therefore, risk factors other than IOP are involved in the pathogenesis of glaucoma. Since the retina has a very high oxygen-demand, decreased autoregulation and a fluctuating oxygen supply to the retina have been linked to glaucomatous neurodegeneration. To assess the significance of these mechanisms, we have utilized a human experimental model, in which we stress participants with a fluctuating oxygen supply. Levels of oxidative stress molecules, antioxidants, and lipid mediators were measured in the plasma. Patients with NTG, OHT, and control subjects were found to have similar levels of oxidative stress markers. In contrast, patients with OHT had a higher level of total antioxidant capacity (TAC) and pro-homeostatic lipid mediators. Thus, we suggest that OHT patients manage fluctuating oxygen levels more efficiently and, thus, are less susceptible to glaucomatous neurodegenerations, due to enhanced systemic antioxidant protection.

## 1. Introduction

Glaucoma is one of the most frequent causes of irreversible blindness worldwide [1], and the number of glaucoma patients will double by 2040 as a result of the growing elderly population [2]. Glaucoma is defined as a progressive loss of the innermost nerve cells of the retina, the retinal ganglion cells, with a simultaneous characteristic loss of the peripheral visual field [1,3]. A major risk factor for the development of glaucoma is elevated intraocular pressure (IOP), and the sole treatment strategies currently available are IOP-lowering medical or surgical treatments [4,5]. Although IOP-lowering treatment strategies often slow the rate of glaucoma progression, far too many go blind despite well-treated IOPs [4,5,6,7]. In this context, a Swedish study has shown that 42% of diagnosed glaucoma patients lose sight in one eye, while 15% end up blind [4].

The most common form of glaucoma in the western world is primary open-angle glaucoma (POAG). POAG can be subdivided into two clinical phenotypes depending on the IOP. Up to 50% of POAG patients have glaucomatous neurodegeneration despite an IOP within the normal range, denoted normal-tension glaucoma (NTG), while the rest have increased IOP, denoted high-tension glaucoma (HTG) [8,9]. In addition to these clinical subgroups within POAG, there is a group of patients with elevated IOP, but no signs of glaucomatous neurodegeneration, patients with ocular hypertension (OHT). The apparent resistance towards increased IOP in OHT patients formed the basis of the present study.

It is recognized that glaucoma is a multifactorial condition with a number of competing risk factors [1,10]. The susceptibility towards the different risk factors is most probably different between patients. Thus, IOP may be a major risk factor in some patients, whereas other risk factors may be more significant in other patients. A widely suggested IOP-independent risk factor is dysfunctional retinal autoregulation [11,12]. Such dysfunctional retinal autoregulation will cause a fluctuating oxygen supply to the retina, thereby increasing the level of reactive oxygen species (ROS) [13,14]. In accordance with this, elevated levels of oxidative stress have been observed in POAG patients [10,15,16,17,18,19].

In the present study, the levels of oxidative stress, as well as antioxidants, were investigated in patients with glaucomatous neurodegeneration and compared to patients with OHT. Patients with NTG were selected based on a hypothetical assumption that these patients are more vulnerable towards other risk factors than IOP, compared to HTG patients, and thus, the most distant phenotype from OHT patients with high IOP but no signs of glaucomatous neurodegeneration. In addition to examining baseline levels of oxidative stress molecules and antioxidants, the response to altered oxygen supply in the test groups was investigated. We imagine that changes in oxidative stress may be less in OHT patients compared to NTG patients during a fluctuating oxygen supply. For this purpose, all tested people were exposed to two hours of hypoxia, followed by 30 min of reperfusion in a full monitor setup. Blood samples were taken before, during, and after hypoxia, and systemic levels of oxidative stress markers, as well as antioxidants, were measured by means of multiple assays. Since polyunsaturated fatty acids (PUFAs), including the ω-3 fatty acid docosahexaenoic acid (DHA) and the ω-6 fatty acid arachidonic acid, can act as antioxidants [20,21,22,23,24,25,26,27,28], we further performed liquid chromatography with tandem mass spectrometry (LC-MS/MS)-based lipidomic analysis to investigate levels of lipid peroxidation and pro-homeostatic lipid mediators in plasma at baseline and in response to a fluctuating oxygen supply.

In summary, by utilizing a human experimental model, the oxidative–antioxidative balance was assessed in response to a fluctuating oxygen supply in patients with glaucomatous neurodegeneration compared to patients with OHT, a condition with an apparent resistance towards glaucoma despite high IOP. The presented results suggest that patients with NTG might suffer from oxidative stress due to an ineffective antioxidant defense compared to OHT patients. In conclusion, our results indicate that OHT patients’ apparent resistance to glaucomatous neurodegeneration may be due to a higher level of antioxidant capacity.

## 2. Experimental Section

This study is an interventional case-control study that followed the tenets of the Declaration of Helsinki of 1975 with ethical approval from the National Committee on Health Research Ethics (Project identification code: H-2-2014-060, 1 February 2015). Written informed consent was obtained from each participant for inclusion before enrolment in the study. Additionally, participants gave oral consent after having the extent of the study explained verbally.

### 2.1. Power Calculations

A direct power calculation based on former studies was not possible as previous studies only included one test group [29,30,31]. Furthermore, we were unable to perform a power calculation based on previously published data [32] as two study groups were included in this study. Power and sample size calculations were therefore performed based on a previous study [33] on arterial vessel diameters as a parallel project that measured retinal vessel diameters during hypoxia. In one study [33], the mean arterial vessel diameter was found to be 204.4 μm with a standard deviation of 18.6. With a power of 80%, a *p*-value of 0.05, and an expected variation in diameters of 12%, a total of at least nine participants in each group was required.

### 2.2. Recruitment Process

**Figure A1:**
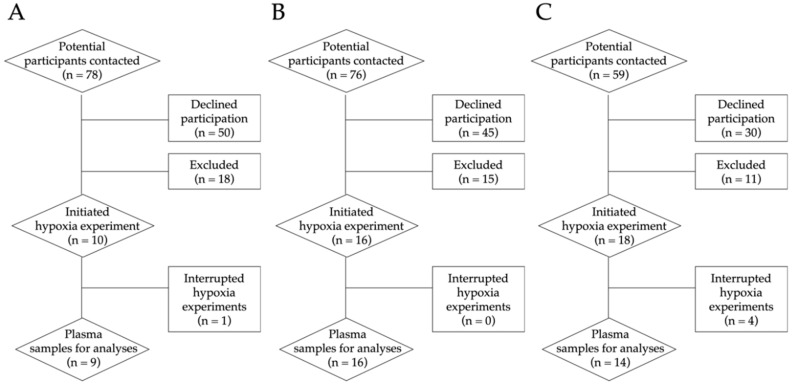
Flow chart of recruitment process for the human experimental model. Out of 213 potential participants allocated into 78 OHT patients, 76 NTG patients, and 59 controls, plasma samples were collected from 9 patients with OHT (**A**), 16 patients with NTG (**B**), and 14 controls (**C**) that completed the experimental setup.

A total of 39 individuals, allocated into 16 patients with NTG, 9 patients with OHT, and 14 age-matched healthy controls were recruited for the study from May 2015 to April 2018. The recruitment process of the study groups is outlined in Figure A1 of Appendix A. Test groups for analyses of oxidative stress and antioxidant markers, as well as lipidomics, overlap to such a degree that the results are comparable. Demographics and clinical data of the study groups match. Patients with NTG and OHT were recruited from the Department of Ophthalmology, Rigshospitalet–Glostrup, Denmark, or from general ophthalmologists. Controls were recruited either from general ophthalmologists or as spouses to patients with NTG or OHT.

All patients with NTG and OHT had a full eye exam performed by a glaucoma specialist. Age-matched controls had an eye exam performed by either a glaucoma specialist or a general ophthalmologist. All patients with NTG were examined within three months before inclusion. At least three ophthalmologists agreed on the diagnosis, and at least six perimetries were performed before inclusion. Patient reports confirmed that IOP measurement had never been measured above 21 mmHg. The number of IOP measurements before inclusion varied in the NTG group, but all patients had information on IOP prior to treatment, and at least six IOP measurements were performed before enrollment in the study. Inclusion criteria for patients with NTG were as follows: untreated IOP below 21 mmHg measured at different times of the day (8 a.m.–5 p.m.); open angles determined by gonioscopy; optic disc cupping, characterized by a violated ISNT rule (normal eyes show a characteristic configuration for disc rim thickness of inferior ≥ superior ≥ nasal ≥ temporal); glaucomatous visual field loss identified by Humphrey or Octopus perimetry, and significant nerve fiber loss detected by ocular coherence tomography (OCT) [32]. Inclusion criteria for OHT patients were as follows: untreated IOP above 24 mmHg measured at different times of the day (8 a.m.–5 p.m.); open angles determined by gonioscopy; no signs of optic disc cupping; normal visual fields, and normal OCT [32]. Exclusion criteria for all participants were as follows: a medical history of ocular trauma; competing eye conditions other than glaucoma affecting the optic nerve; significant systemic diseases, i.e., dysregulated hypertension, heart failure, hypercholesterolemia, diabetes mellitus, autoimmune diseases, and previous cerebral infarct or hemorrhage; subjects who could not contribute with the experimental setup; subjects under 50 years of age, and subjects who smoked at the time of inclusion. To check whether participants met the inclusion and exclusion criteria, medical history and a list of medications were collected from each participant. Moreover, demographic information on age, height, weight, body mass index (BMI), and gender were collected on the day of enrolment (Table 1). General ophthalmic data, including IOP and the mean defect (MD) from a reliable visual field exam, were obtained (Table 2).

### 2.3. Hypoxia Model

**Figure 1:**
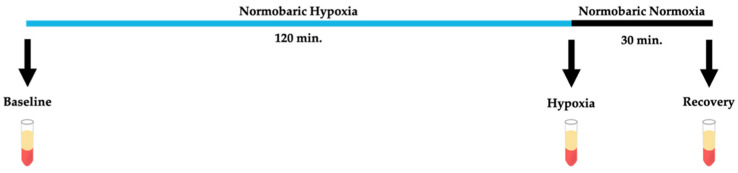
Time course of experimental setup for hypoxic intervention. Participants were exposed to 120 min of normobaric hypoxia followed by 30 min of normobaric normoxia. Each arrow indicates a time point (“Baseline”, “Hypoxia”, and “Recovery”) where peripheral blood samples were withdrawn from a forearm vein.

Participants fasted 10 h prior to the initiation of the experimental setup. Before hypoxic intervention, participants received pupil dilating drops. The intervention with hypoxia has previously been described by Vohra et al. [32]. Briefly, each participant was exposed to normobaric hypoxia for two hours through a tightly fitting facemask attached to a non-breathable valve followed by normobaric normoxia by normal breathing in a supine position. By using a non-breathable valve, we assured that participants would not breathe a hypercapnic gas. The facemask was connected using a Y-piece to a Douglas bag containing a humidified gas mixture of 10% oxygen and 90% nitrogen. Our model is safely conducted in a laboratory setting with continuous monitoring of heart rate (HR), blood pressure and arterial oxygen saturation. In case of any malaise, the experimental setup was immediately interrupted. None of our subjects showed any sign of intolerance to hypoxia and no elevation of blood pressure at rest over 20 mmHg during hypoxia was observed. Along with other laboratories worldwide, we have used this model in numerous studies and regard it as safe and reproducible. In general, the occurrence of optic neuropathy has been reported at high altitude, but only after prolonged exposure to very high altitudes above 5000 m and in combination with exercise, or in one case report in combination with increased blood pressure and in combination with exercise. In the present hypoxic model, 10% oxygen equals the environment at high trekking routes in the Alps, annually visited by thousands of people. With the current use of a limited time of two hours together with sedentary position, controlled blood pressure and no subjective discomfort, it is very unlikely that optic neuropathy could be induced. Rather, it serves the purpose of inducing a safe and reproducible challenge to short-term hypoxia, used by several groups to unveil pathological conditions. In summary, we estimate that the risk of optic neuropathy in our stringently monitored experimental setup is highly unlikely. At three defined time points: before initiation of hypoxia (“baseline”), during hypoxia (“hypoxia”), and after 30 min of normoxia (“recovery”), blood samples were withdrawn from a forearm vein by routine vein puncture (Figure 1). At each time point, 3 × 6 mL whole blood was taken in EDTA tubes, inverted several times, and kept on ice. Within one hour from sampling, whole blood was centrifuged in EDTA tubes at 4000 RPM for 10 min at 4 °C, after which plasma was aliquoted to Eppendorf tubes with a Pasteur pipette and stored at −80 °C for further analyses.

### 2.4. Antioxidant–Oxidant Status

Markers for oxidative stress and antioxidant defense were measured by colorimetric methods in plasma from 14 controls, 16 patients with NTG, and 9 OHT patients.

#### 2.4.1. Total Antioxidant Capacity Analysis

At the time of total antioxidant capacity (TAC) analysis, plasma was thawed on ice. Plasma was diluted 1:10 in Assay buffer, and TAC level was expressed as molar (M). TAC levels were determined using a colorimetric assay kit (Cayman Chemical, Ann Arbor, MI, USA; Item no: 709001, Batch: 0530656, Nashville, TN, USA) according to the manufacturer’s instructions.

#### 2.4.2. Superoxide Dismutase 3 Analysis

At the time of superoxide dismutase 3 (SOD3) analysis, plasma was thawed on ice. Plasma was diluted 1:5 in sample buffer, and SOD3 activity was expressed as U/mL. SOD3 activity was determined using a colorimetric assay kit (Cayman Chemical, LOT no: 706002, Ann Arbor, MI, USA) according to the manufacturer’s instructions.

#### 2.4.3. Malondialdehyde Analysis

Samples were thawed on ice before malondialdehyde (MDA) analysis. Plasma was analyzed undiluted, and MDA level was expressed as mmol/L. MDA levels were determined by a colorimetric trichloroacetic acid method involving thiobarbituric acid adduct formation (Cayman Chemical, Item no: 700870, USA) according to the manufacturer’s instructions.

#### 2.4.4. Free Radical Analysis

ROS/reactive nitrogen species (RNS) analysis was performed after thawing on ice. Plasma was diluted 1:2 in PBS, and ROS/RNS level was expressed as μM. ROS/RNS levels were determined using a colorimetric assay kit “OxiSelect^TM^ In Vitro ROS/RNS Assay Kit (Green Fluorescence)” (Cell Biolabs, San Diego, CA, USA; Catalog no: STA-347, LOT: 7081341, Torrance, CA, USA) according to the manufacturer’s instructions.

### 2.5. Lipidomics

Lipidomic analyses were performed on plasma samples from 9 controls, 9 patients with NTG, and 9 patients with OHT.

#### 2.5.1. Lipid Extraction

A modified Bligh–Dyer method [34] was used for lipid extraction. In short, plasma samples were mixed with 2.5× volumes of cold MeOH and put on ice for 30 min for protein precipitation. The samples were centrifuged, and supernatants were collected into glass tubes. 1.25 volumes of CHCl_3_ were added, followed by the 5 μL of an internal standard mixture (LTB4-d_4_, PGD2-d_4_, 15-HETE-d_8_, AA-d_8_, and EPA-d_5_). After sonicated in the water bath with ice for 30 min, the samples were stored at −80 °C overnight.

The samples were added to another 1.25 volume of CHCl_3_, and 1.25 volumes of H_2_O (adjusted to pH 3.5 with HCl) for the phase separation. The upper phase was removed, and the lipid phase was dried under nitrogen flow. The lipid phase was re-constructed in 30 μL of MeOH:H_2_O 1:1 for mass spectrometric measurements.

#### 2.5.2. LC-MS/MS

A Xevo TQ-S mass spectrometer equipped with Acquity I Class UPLC (Waters Corporation, Milford, MA) was used for lipidomic analyses. Separation of fatty acids and their derivatives was performed on a CORTECS C18 column (2.7 μm particle size, 4.6 mm × 100 mm i.d.) (Waters Corporation, Milford, MA, USA) using the following gradient of 45% of solvent A (H_2_O + 0.01% acetic acid) and 55% of solvent B (MeOH + 0.01% acetic acid) and a flow rate of 0.6 mL/min: 0 min, 55% B, 10 min, 85% B, 18 min, 98% B, 20 min, 98% B, 30 min, 55% B. The capillary voltage was −2.5 kV, desolvation temperature was 600 °C, desolvation gas flow was 1100 L/Hr, cone gas was 150 L/Hr, and nebulizer pressure was 7.0 bar with a source temperature of 150 °C. MassLynx ver. 4.1 software was used for the operation and recording of the data. Data were expressed as the change in the relative abundance of pro-homeostatic lipid mediators normalized with the internal standards.

### 2.6. Statistical Analysis

Data are presented as mean ± SEM. All data in tables are presented as mean ± SD. Data were analyzed with Graphpad Prism 8 software (Graphpad Software, version 8.1.2, San Diego, CA, USA). Outliers were detected with Grubb’s test (Graphpad Software, Quickcalcs, San Diego, CA, USA). One-way ANOVA was performed to analyze data from multiple groups or multiple time points, followed by multiple comparisons test using Tukey’s multiple comparisons test. Two-way ANOVA was performed to analyze systemic vital parameters followed by Tukey’s multiple comparisons test. *p* < 0.05 was considered statistically significant.

## 3. Results

### 3.1. Demographics and Ophthalmological Characteristics

Age, height, weight, BMI, and gender distribution were not significantly different between the test groups (Table 1). With regard to the ophthalmological characteristics, significant differences were observed as expected. Thus, IOP was significantly higher in OHT patients on both eyes compared to controls (oculus dexter (OD): *p* = <0.0001; oculus sinister (OS): *p* = <0.0001) and patients with NTG (OD: *p* = <0.0001; OS: *p* = <0.0001). No significant differences in IOP were observed between controls and patients with NTG (Table 2). MD values were significantly higher in patients with NTG in both eyes compared to controls (OD: *p* = 0.0046; OS: *p* = 0.00050) and OHT patients (OD: *p* = 0.038; OS: *p* = 0.0047). No significant differences in MD values were identified between controls and OHT patients (Table 2).

### 3.2. Stress Response Regarding Systemic Vital Parameters

**Figure 2:**
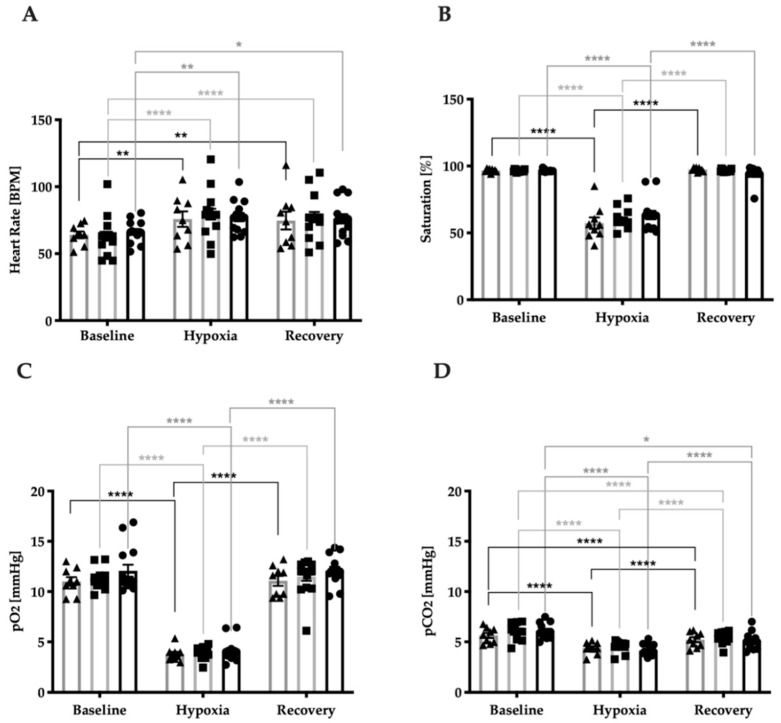
Stress responses of systemic vital parameters in patients with OHT (▲), patients with normal-tension glaucoma (NTG) (∎), and controls (●). Heart rate (HR) (**A**) was measured by a Nexfin monitor. Saturation (SAT) (**B**), partial pressure of oxygen (pO_2_) (**C**), and partial pressure of carbon dioxide (pCO_2_) (**D**) were detected by the ABL8000Flex in arterial blood gas. Data are presented as mean ± SEM. Two-way ANOVA with Tukey’s multiple comparisons test. * *p* < 0.05; ** *p* < 0.01; **** *p* < 0.0001.

No statistically significant differences were identified in HR, saturation (SAT), partial pressure of oxygen (pO_2_), or partial pressure of carbon dioxide (pCO_2_) between the studied groups indicating that all three groups responded similarly to the hypoxic intervention regarding systemic vital parameters. All systemic vital parameters were significantly regulated during hypoxic intervention (two-way ANOVA, Figure 2A; F(2, 72) = 30.92, *p* < 0.0001; Figure 2B; F(2, 72) = 421.1, *p* < 0.0001; Figure 2C; F(2, 72) = 446.3, *p* < 0.0001; Figure 2D; F(2, 72) = 175.0, *p* < 0.0001). HR increased from baseline to hypoxia and recovery in controls (*p* = 0.0060; *p* = 0.010), NTG patients (*p* = 0.0010; *p* = 0.0042), and OHT patients (*p* = <0.0001; *p* = <0.0001) (Figure 2A). Venous SAT decreased from baseline to hypoxia and increased from hypoxia to recovery in controls (*p* = <0.0001; *p* = <0.0001), NTG patients (*p* = <0.0001; *p* = <0.0001), and OHT patients (*p* = <0.0001; *p* = <0.0001) (Figure 2B). pO_2_ decreased from baseline to hypoxia and increased from hypoxia to recovery in controls (*p* = <0.0001; *p* = <0.0001), NTG patients (*p* = <0.0001; *p* = <0.0001), and OHT patients (*p* = <0.0001; (*p* = <0.0001) (Figure 2C). pCO_2_ decreased from baseline to hypoxia and increased from hypoxia to recovery in controls (*p* = <0.0001; *p* = <0.0001), NTG patients (*p* = <0.0001; *p* = <0.0001), and OHT patients (*p* = <0.0001; *p* = <0.0001). Additionally, pCO_2_ decreased from baseline to hypoxia in controls (*p* = <0.0001), patients with NTG (*p* = <0.0001), and patients with OHT (*p* = 0.037) (Figure 2D).

### 3.3. Increased Antioxidant Capacity in Patients with OHT Compared to NTG and Controls

**Figure 3:**
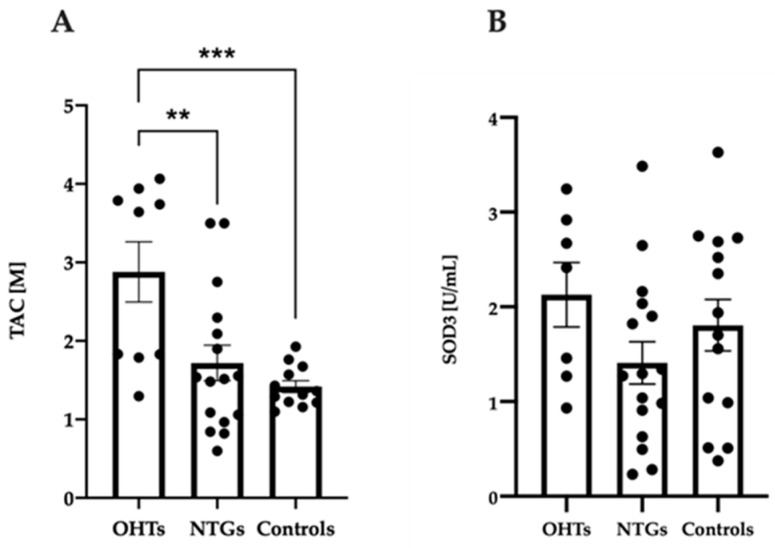
Total antioxidant capacity (TAC) level (**A**) and superoxide dismutase 3 (SOD3) activity (**B**) at baseline in patients with OHT, patients with NTG, and controls. TAC level and SOD3 activity were measured in plasma by colorimetric methods. Data are presented as mean ± SEM. One-way ANOVA with Tukey’s multiple comparisons test. ** *p* < 0.01; *** *p* < 0.001.

**Figure A2:**
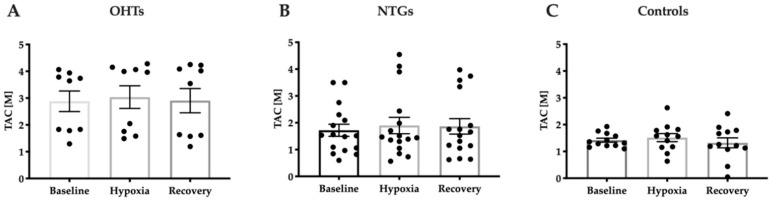
TAC level during hypoxic exposure in patients with OHT (**A**), patients with NTG (**B**), and controls (**C**). TAC was measured in plasma by a colorimetric method. Data are presented as mean ± SEM. One-way ANOVA with Tukey’s multiple comparisons test.

To investigate possible deviations in the systemic antioxidant defense of blood samples from patients with OHT, NTG and controls, the overall antioxidant capacity was evaluated from plasma collected at baseline. A colorimetric method that assessed the general ability of a blood sample to counteract an oxidative reaction was chosen. Patients with OHT expressed significantly higher levels of TAC at baseline compared to the other groups (Figure 3A; one-way ANOVA, F(2, 34) = 8.60, *p* = 0.0010). The TAC level in OHT patients was elevated compared to both patients with NTG (*p* = 0.0056) and controls (*p* = 0.0010). No significant difference was observed between patients with NTG and controls (Figure 3A). TAC levels in plasma samples collected at the two other time points, hypoxia and recovery, were further evaluated to test whether any of the groups regulated levels of TAC during hypoxia. There was no significant regulation of TAC due to neither hypoxia nor reperfusion in any of the test groups (one-way ANOVA, Appendix B, Figure A2A; F(2, 24) = 0.041, *p* = 0.96; Figure A2B; F(2, 45) = 0.12, *p* = 0.89, Figure A2C; F(2, 33) = 0.46, *p* = 0.64).

**Figure A3:**
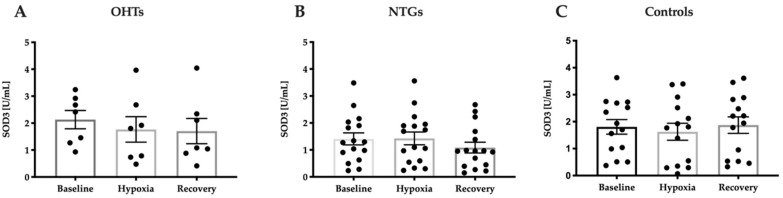
SOD3 activity during hypoxic exposure in patients with OHT (**A**), patients with NTG (**B**), and controls (**C**). SOD3 activity was measured in plasma by a colorimetric method. Data are presented as mean ± SEM. One-way ANOVA with Tukey’s multiple comparisons test.

Next, we sought to investigate which specific antioxidants are responsible for this increased capacity in OHT patients. In this context, the same plasma samples were analyzed for the activity of the most prominent extracellular antioxidant enzyme SOD3. No significant differences were identified between the test groups (Figure 3B; one-way ANOVA, F(2, 34) = 1.59, *p* = 0.22). The activity of SOD3 in plasma samples collected at hypoxia and recovery was also evaluated to test whether the three groups deviated from each other regarding regulation of this important antioxidant enzyme. SOD3 was not significantly regulated in any of the test groups during hypoxic intervention (one-way ANOVA, Appendix B, Figure A3A; F(2, 18) = 0.29, *p* = 0.75; Figure A3B; F(2, 45) = 0.76, *p* = 0.48; Figure A3C; F(2, 39) = 0.18, *p* = 0.83).

### 3.4. Oxidative Stress in Study Groups

**Figure 4:**
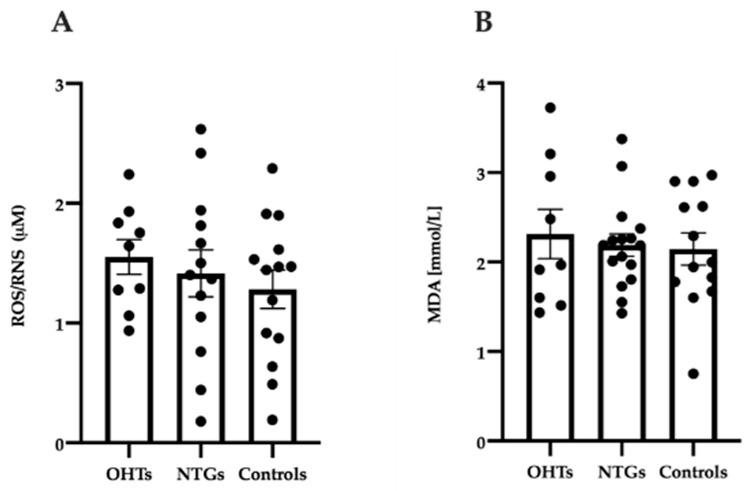
Overall oxidative stress level measured by reactive oxygen species (ROS)/reactive nitrogen species (RNS) abundance (**A**) and lipid peroxidation reflected by malondialdehyde (MDA) abundance (**B**) at baseline in patients with OHT, patients with NTG, and controls. ROS/RNS and MDA level were measured in plasma by colorimetric methods. Data are presented as mean ± SEM. One-way ANOVA with Tukey’s multiple comparisons test.

**Figure A4:**
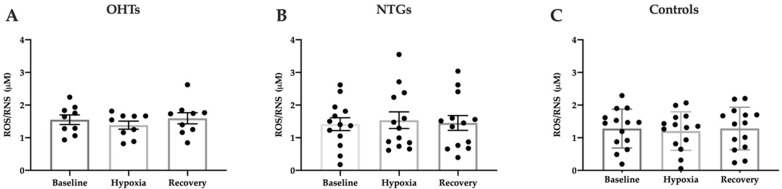
Overall oxidative stress level measured by ROS/RNS abundance during hypoxic exposure in patients with OHT (**A**), patients with NTG (**B**), and controls (**C**). ROS/RNS was measured in plasma by a colorimetric method. Data are presented as mean ± SEM. One-way ANOVA with Tukey’s multiple comparisons test.

To evaluate if the overall levels of oxidative stress were significantly different between test groups, a colorimetric method that evaluates the total plasma levels of free radicals, ROS and RNS, was used to get an overview of the antioxidant-oxidant status of the collected blood samples. No significant differences were observed between the studied groups (Figure 4A; one-way ANOVA, F(2, 33) = 0.55, *p* = 0.58). ROS/RNS levels were further analyzed in plasma samples obtained during hypoxia and recovery. No significant differences were identified throughout the experiment for any of the test groups included in this study (one-way ANOVA, Appendix B, Figure A4A; F(2, 24) = 0.57, *p* = 0.58; Figure A4B; F(2, 36) = 0.074, *p* = 0.93; Figure A4C; F(2, 39) = 0.075, *p* = 0.93).

**Figure A5:**
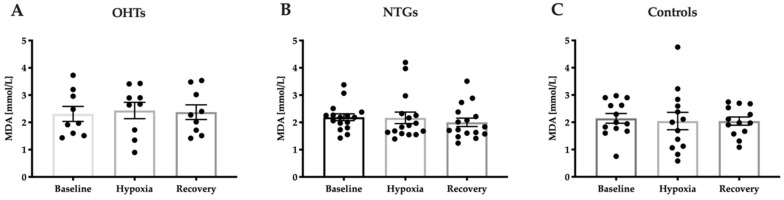
Lipid peroxidation reflected by MDA abundance during hypoxic exposure in patients with OHT (**A**), patients with NTG (**B**), and controls (**C**). MDA was measured in plasma by a colorimetric method. Data are presented as mean ± SEM. One-way ANOVA with Tukey’s multiple comparisons test.

All cellular components are potential subjects for oxidative damage. One of the major targets are lipids through lipid peroxidation that affects the cellular membrane causing changes to the surface and organelles of the cell. Lipid peroxides are unstable molecules that degrade into complex series of molecules, including reactive carbonyl compounds, such as MDA. We thus investigated the level of MDA in plasma samples. No significant differences were identified between patients with NTG, OHT patients, and controls (Figure 4B; one-way ANOVA, F(2, 35) = 0.19, *p* = 0.83). Furthermore, MDA levels were analyzed in plasma samples taken at hypoxia and recovery. No significant differences were identified throughout the experiment for any of the test groups included in this study (one-way ANOVA, Appendix B, Figure A5A; F(2, 24) = 0.048, *p* = 0.95; Figure A5B; F(2, 45) = 0.37, *p* = 0.69; Figure A5C; F(2, 36) = 0.065, *p* = 0.94).

### 3.5. Pro-Homeostatic Lipid Mediators Correlate with Antioxidant Capacity

**Figure 5:**
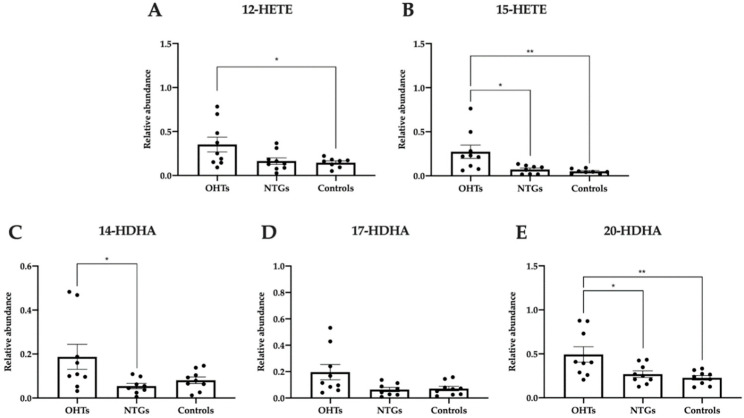
Relative abundance of pro-homeostatic lipid mediators at baseline in patients with OHT, patients with NTG, and controls measured in plasma by liquid chromatography with tandem mass spectrometry (LC-MS/MS). The metabolites of arachidonic acid, 12- hydroxyeicosatetraenoic acids (-HETE) (**A**) and 15-HETE (**B**), and oxygenation products of docosahexaenoic acid (DHA), 14-hydroxydocosahexaenoic acids (-HDHA) (**C**), 17-HDHA (**D**), and 20-HDHA (**E**) were measured. Data are presented as mean ± SEM. One-way ANOVA with Tukey’s multiple comparisons test. * *p* < 0.05; ** *p* < 0.01.

To further investigate the antioxidant–oxidant status, LC-MS/MS-based lipidomic analysis of lipids with potential antioxidant bioactivity was performed. Lipidomic analyses showed that 12-hydroxyeicosatetraenoic acids (-HETE), 15-HETE, 14-hydroxydocosahexaenoic acids (-HDHA), and 20-HDHA were increased in patients with OHT compared to NTG patients and/or controls at baseline (one-way ANOVA, Figure 5A; F(2, 23) = 4.16, *p* = 0.029; Figure 5B; F(2, 22) = 6.70, *p* = 0.0053; Figure 5C; F(2, 23) = 3.87, *p* = 0.036; Figure 5D; F(2,23) = 4.00, *p* = 0.032; Figure 5E; F(2, 24) = 6.31, *p* = 0.0063). Patients with OHT had a 12-HETE level that was elevated compared to controls (*p* = 0.045) and a 15-HETE level that was higher than both controls (*p* = 0.0091) and patients with NTG (*p* = 0.019). Additionally, OHT patients had increased levels of 14-HDHA compared to NTG patients (*p* = 0.042) and 20-HDHA compared to controls (*p* = 0.0081) and patients with NTG (*p* = 0.027). No significant differences were identified between groups regarding 17-HDHA.

**Figure 6:**
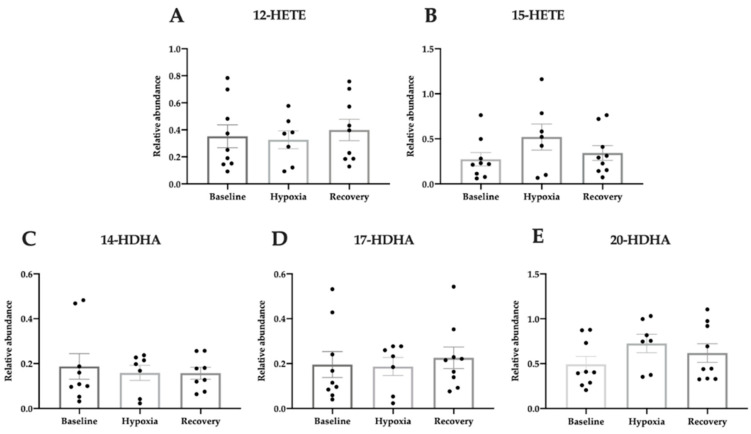
Relative abundance of pro-homeostatic lipid mediators during hypoxic exposure in patients with OHT measured in plasma by LC-MS/MS. The metabolites of arachidonic acid, 12-HETE (**A**) and 15-HETE (**B**), and oxygenation products of DHA, 14-HDHA (**C**), 17-HDHA (**D**), and 20-HDHA (**E**) were measured. Data are presented as mean ± SEM. One-way ANOVA with Tukey’s multiple comparisons test.

Further lipidomic analyses on blood samples taken during hypoxia and reperfusion were performed. In patients with OHT, none of the investigated lipid mediators were significantly regulated during hypoxic intervention (one-way ANOVA, Figure 6A; F(2, 22) = 0.21, *p* = 0.81; Figure 6B; F(2, 22) = 1.54, *p* = 0.24; Figure 6C; F(2, 21) = 0.16, *p* = 0.85; Figure 6D; F(2, 22) = 0.16, *p* = 0.85; Figure 6E; F(2, 22) = 1.33, *p* = 0.28).

**Figure 7:**
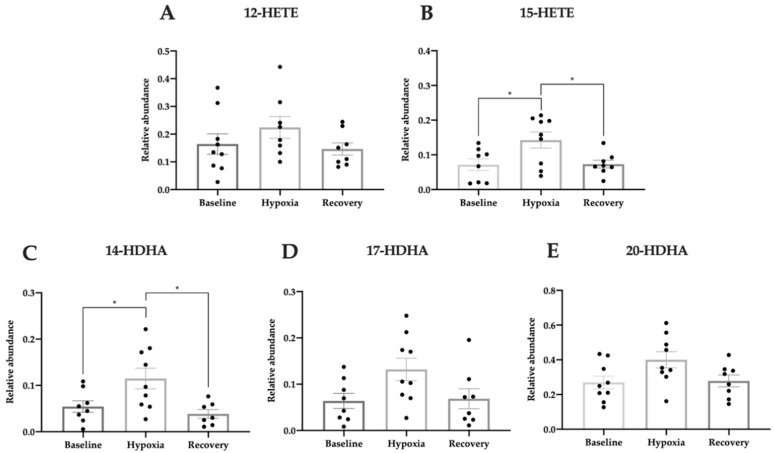
Relative abundance of pro-homeostatic lipid mediators during hypoxic exposure in patients with NTG measured in plasma by LC-MS/MS. The metabolites of arachidonic acid, 12-HETE (**A**) and 15-HETE (**B**), and oxygenation products of DHA, 14-HDHA (**C**), 17-HDHA (**D**), and 20-HDHA (**E**) were measured. Data are presented as mean ± SEM. One-way ANOVA with Tukey’s multiple comparisons test. * *p* < 0.05.

Patients with NTG regulated 15-HETE and 14-HDHA in response to hypoxic exposure (one-way ANOVA, Figure 7A; F(2, 22) = 1.40, *p* = 0.27; Figure 7B; F(2, 22) = 5.09, *p* = 0.015; Figure 7C; F(2, 21) = 5.84, *p* = 0.0096; Figure 7D; F(2, 22) = 3.28, *p* = 0.057; Figure 7E; F(2, 23) = 3.40, *p* = 0.051). The 15-HETE level increased from hypoxia to recovery (*p* = 0.029) and decreased thereafter from hypoxia to recovery (*p* = 0.034). 14-HDHA also increased during hypoxic intervention (*p* = 0.043) and decreased in the recovery phase (*p* = 0.013). None of the other lipid mediators were significantly regulated in patients with NTG in response to hypoxia.

**Figure 8:**
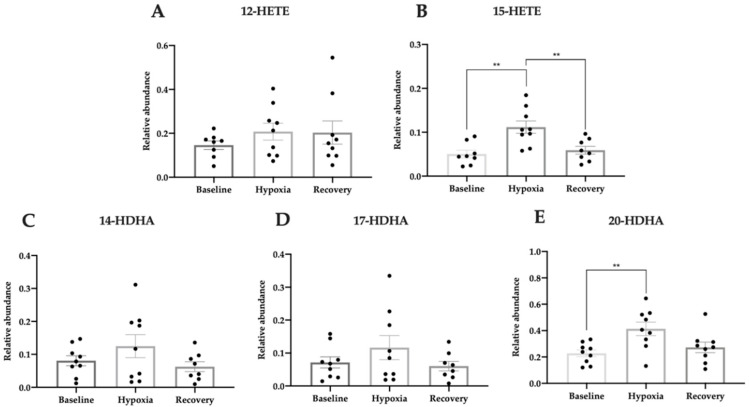
Relative abundance of pro-homeostatic lipid mediators during hypoxic exposure in controls measured in plasma by LC-MS/MS. The metabolites of arachidonic acid, 12-HETE (**A**) and 15-HETE (**B**), and oxygenation products of DHA, 14-HDHA (**C**), 17-HDHA (**D**), and 20-HDHA (**E**) were measured. Data are presented as mean ± SEM. One-way ANOVA with Tukey’s multiple comparisons test. ** *p* < 0.01.

In controls, 15-HETE and 20-HDHA were significantly regulated during hypoxic exposure (one-way ANOVA, Figure 8A; F(2, 23) = 0.69, *p* = 0.51; Figure 8B; F(2, 22) = 9.14, *p* = 0.0013; Figure 8C; F(2, 23) = 1.72, *p* = 0.20; Figure 8D; F(2, 23) = 1.36, *p* = 0.28; Figure 8E; F(2, 24) = 5.80, *p* = 0.0088). The 15-HETE level increased from baseline to hypoxia (*p* = 0.0021) and decreased from hypoxia to recovery (*p* = 0.0077), while 20-HDHA increased during hypoxia (*p* = 0.0089). None of the other lipid mediators were significantly regulated in controls in response to hypoxia.

## 4. Discussion

A link between oxidative stress and glaucomatous neurodegeneration has been suggested [10,15,17,35,36,37,38,39,40,41,42,43,44]. In this study, we show increased baseline levels of TAC in patients with OHT. Moreover, we report an increased abundance of oxidation products of DHA and derivatives of arachidonic acid, i.e., lipid mediators with antioxidative functions in OHT patients. In our human experimental model, we find significant regulations of pro-homeostatic lipid mediators in response to a fluctuating oxygen supply in patients with NTG and controls, whereas there was no significant regulation in patients with OHT. Although increased levels of oxidative stress were not detected in any of the test groups, neither at baseline, nor in response to a fluctuating oxygen supply, our results suggest a relationship between increased antioxidant capacity and resistance towards glaucomatous neurodegeneration in patients with OHT. To our knowledge, this is the first study evaluating oxidative stress and antioxidative mediators in patients with NTG and OHT during oxygen stress.

Decreased TAC in serum from glaucoma patients compared with control subjects [15,17,36,39,41,45,46,47] and cataract patients [42,48] has been reported. Such findings indicate that glaucoma patients have a reduced capacity to cope with increasing oxidative stress during aging [49,50]. One previous study has found contradicting results with increased levels of TAC in patients with NTG compared to controls [44]. We could not confirm any differences in TAC between patients with NTG and controls. Our present finding of increased TAC in plasma from patients with OHT compared to both patients with NTG and controls indicates a critical role of antioxidant capacity in preventing glaucomatous neurodegeneration. In support of our results, a study by Lascaratos et al. has shown enhanced systemic mitochondrial efficiency in patients with OHT compared to patients with NTG and controls in isolated lymphocytes from peripheral blood samples [1].

To explore specific antioxidants, we examined the activity of the extracellular antioxidant, SOD3. Our results did not identify any significant differences in SOD3 between the tested groups. Generally, there are discrepancies between studies on SOD levels in glaucoma patients compared to controls. Whereas some studies have reported decreased SOD in serum from glaucoma patients compared with controls [17,39], other studies have found increased SOD in glaucoma patients compared to cataract patients in aqueous humor [45,51,52] and red blood cells (RBCs) [3]. To our knowledge, only one study has explored SOD levels in patients with OHT. In this study, patients with NTG had elevated levels of SOD in isolated lymphocytes compared to patients with OHT [1]. The contradicting results may be explained by the different SOD isoenzymes of blood cells, aqueous humor, serum, and plasma. Extracellular SOD3 is more relevant in serum and plasma, while the cytosolic SOD1 and mitochondrial SOD2 are more relevant in blood cells. The expression and activity of SOD isoenzymes are known to be highly tissue- and compartment-specific, which should be taken into account when comparing observations. Based on the studies described above, it seems that changes during glaucomatous neurodegeneration are similar in aqueous humor and RBCs but different in serum. The lack of a significant increase in SOD3 of patients with NTG or an elevation during hypoxic exposure in the included groups may though also indicate an exhaustion of the antioxidant defense in plasma. It leaves us with an unanswered question about the involvement of SOD3 in the vulnerability towards glaucomatous neurodegeneration and in the antioxidant defense of OHT.

To further investigate the oxidative–antioxidant balance, we investigated lipid peroxidation. Previous studies have reported increased MDA in serum [15,38,39,41,43] and RBCs [3] in patients with glaucoma. Increased MDA in the aqueous humor has also been observed in glaucoma patients compared to patients with cataract [48,51]. Results from the current study did not identify significant differences in free radicals or MDA levels between patients with NTG, patients with OHT, and controls. Overall, no direct evidence for increased levels of oxidative stress between the test groups was found. Thus, we could not confirm any of these previous findings regarding the glaucoma patient group. Even though we did not detect higher levels of free radicals or lipid peroxidation in either of the analyzed groups, further studies are needed to rule out these processes in the pathology of NTG and OHT. The increased levels of TAC in OHT patients continue to indicate that some processes of oxidative stress are actively counteracted by antioxidant defenses, and thereby also could be involved in the clinical phenotypes of OHT and NTG. Therefore, it remains to be elucidated whether levels of other enzymes or molecules of oxidative stress-related processes are altered in patients with OHT and patients with NTG in comparison to controls.

To examine the high antioxidant defense observed in patients with OHT, we chose to investigate pro-homeostatic lipid mediators derived from PUFAs [20,21,53,54,55,56,57]. The biosynthesis of docosanoids from DHA leads to the formation of stable HDHAs, three of which were measured in the current study. Anti-inflammatory properties of all three have been described [23,24,58]. Elevated levels of two of the measured HDHAs were identified in patients with OHT compared to patients with NTG and/or controls, indicating that these pro-homeostatic lipid mediators may contribute to higher antioxidant capacity levels in OHT patients. Arachidonic acid can yield HETEs, of which two were analyzed. Some discrepancies regarding the oxidant or antioxidant effect of these derivatives are found in the literature. A former study has identified neuroprotective effects of both HETEs [59]. Some contradicting studies have suggested that 12-HETE cause mitochondrial dysfunction [60] and that 15-HETE can induce ROS production [61]. Increased levels of both HETEs were identified in the OHT group compared to controls and/or patients with NTG. In line with current results, we believe that these increased levels indicate components of the antioxidant defense in OHT patients. High levels of pro-homeostatic lipid mediators add to the understanding of how patients with OHT might withstand elevated IOP and glaucomatous neurodegeneration.

In the present study, we utilized our human experimental model in which strictly characterized clinical phenotypes, with and without glaucoma, underwent hypoxia followed by a reperfusion period. All groups regulated systemic vital parameters, HR, SAT, pO_2_, and pCO_2_, significantly during the hypoxia model, confirming that the model allowed us to compare both baseline levels and stress responses of the included groups. Levels of lipid mediators, 14-HDHA, 20-HDHA, and 15-HETE, were varying during exposure to fluctuating oxygen supplies in controls and/or patients with NTG, whereas levels remained stable in OHT patients. These results indicate that the naturally occurring high antioxidant defense present in patients with OHT could be enough to tolerate oxygen stress without regulating levels of lipid mediators.

Apart from pro-homeostatic lipid mediators, no other analyzed markers varied significantly with fluctuating oxygen levels. These results might indicate that alterations in the level of enzymes, lipids, and smaller molecules related to oxidative and antioxidative mechanisms in plasma are highly heterogeneous. A measurable oxidative stress response in plasma induced by hypoxia depends on both the duration and intensity of hypoxia [62]. It is possible that some of these molecular changes occur more rapidly or might be a more long-term effect. In our previous study with the same human experimental model we showed changes in plasma levels of amino acids and lactate in response to hypoxia [32]. Another study measured increased markers associated with oxidative stress during normobaric hypoxia with 12.9% oxygen for nine hours [63]. Additional studies have evaluated the effect of hypobaric hypoxia for either four hours [64], 72 h [65], three days [66], or 13 days [67]. Three of these studies investigated oxidative stress and antioxidants in plasma and found a hypoxia-induced increase in oxidative stress [64,65,67] or TAC [64]. Two hours of normobaric hypoxic exposure with 10% oxygen was chosen as the upper limit of acute hypoxia concerning ethics and the comfort of participants. More studies are needed to confirm whether the analyzed oxidative and antioxidant markers are regulated during fluctuating oxygen supplies.

To increase the chance of identifying changes in systemic markers between the analyzed groups, we have carefully considered which clinical phenotypes we should include. Since our goal was to investigate risk factors other than IOP that may play a role in the development of glaucoma, we have chosen to include NTG patients who may be more vulnerable to risk factors other than elevated IOP. To investigate whether some factors, on the other hand, prevent vulnerability to elevated IOP, we have selected the clinical phenotype OHT, characterized by having elevated IOP with no evidence of glaucomatous neurodegeneration. Based on our results, it will be interesting in future studies to investigate whether HTG patients (patients with elevated IOP and glaucomatous neurodegeneration) are similar to OHT or NTG patients to further understand why some are more vulnerable to increased IOP than others.

The retina and aqueous humor, both potentially involved in the pathogenesis of glaucoma, are supplied with nutrients from plasma through the blood–retinal barrier and blood–aqueous barrier that control which and the quantity of substances transferred. It may be, that development of or protection against glaucomatous neurodegeneration is not only affected by systemic levels of free radicals and antioxidants but also by the permeability of the barriers which may be affected by pathological conditions. As the current study focused on a highly complex human experimental setup, it was unfortunately not possible to compare plasma levels of free radicals and antioxidants to intraocular levels. Such comparison would indeed be important in future studies, to investigate whether differences and possible pathological explanations for glaucoma relate only to plasma or can also be found in aqueous humor and ocular tissues as a result of transfer across the barriers.

## 5. Conclusions

The study provides novel insights into possible mechanisms of protection against oxidative stress in patients with OHT and may explain the apparent tolerability to increased IOP in these patients. We show for the first time that patients with OHT may exhibit a superior antioxidant protection due to higher antioxidant capacity and abundance of pro-homeostatic lipid mediators in plasma compared to patients with NTG and controls. The superior antioxidant defense provides potential resistance towards elevated IOP and glaucomatous neurodegeneration by eliminating increases in systemic oxidative stress. Therefore, it is of interest to elucidate in the future whether antioxidants, including pro-homeostatic lipid mediators like HDHAs and HETEs, can become diagnostic biomarkers and open up the exploration of novel therapeutic strategies in glaucoma.

## Figures and Tables

**Table 1:** Participant demographics.

	OHTs	NTGs	Controls
Age (years)	72 ± 4	70 ± 6	66 ± 7
Body Weight (kg)	74.2 ± 10.2	73.9 ± 14.9	74.9 ± 7.4
Height (cm)	172 ± 8	174 ± 10	173 ± 9
BMI ^1^	25 ± 2	24 ± 3	25 ± 3
Gender (F/M)	4/5	8/8	6/8

^1^$BMI=\frac{Body\;Weight\;\left(kg\right)}{Height\;\left(m\right)\times Height\;\left(m\right)}$. BMI: Body mass index; F: Female; M: Male; NTG: Normal-tension glaucoma; OHT: Ocular hypertension.

**Table 2:** General ophthalmological data of participants.

	OHTs	NTGs	Controls
IOP OD (mmHg)	28 ± 3	12 ± 1 ****	13 ± 2 ****
IOP OS (mmHg)	30 ± 4	12 ± 1 ****	13 ± 2 ****
MD OD (dB)	0.84 ± 2.73 ^#^	5.68 ± 6.63	0.96 ± 0.075 ^##^
MD OS (dB)	0.88 ± 4.97 ^##^	8.82 ± 7.87	0.68 ± 0.32 ^###^

IOP: Intraocular pressure: MD: Mean defect; OD: Oculus dexter; OS: Oculus sinister. Different from patients with OHT: **** *p* < 0.0001. Different from patients with NTG: ^#^ *p* < 0.05; ^##^ *p* < 0.01; ^###^ *p* < 0.001.

## Abbreviations

BMI	body mass index
DHA	docosahexaenoic acid
HDHA	hydroxydocosahexaenoic acids
HETE	hydroxyeicosatetraenoic acids
HR	heart rate
HTG	high-tension glaucoma
IOP	intraocular pressure
LC-MS/MS	liquid chromatography with tandem mass spectrometry
M	molar
MD	mean defect
MDA	malondialdehyde
NTG	normal-tension glaucoma
OCT	ocular coherence tomography
OD	oculus dexter
OHT	ocular hypertension
OS	oculus sinister
pCO_2_	partial pressure of carbon dioxide
pO_2_	partial pressure of oxygen
POAG	primary open-angle glaucoma
PUFAs	polyunsaturated fatty acids
RBCs	red blood cells
RNS	reactive nitrogen species
ROS	reactive oxygen species
SAT	saturation
SOD3	superoxide dismutase 3
TAC	total antioxidant capacity

## Appendix A

A total of 213 potential participants allocated into 78 patients with OHT, 76 patients with NTG, and 59 controls were contacted for the study from 2015 to 2018. Out of these, 50 OHT patients, 45 NTG patients, and 30 controls declined to participate in the experimental setup after reading the participant information, while 18, 15, and 11, respectively, were excluded because they did not meet the inclusion and/or exclusion criteria. The human experimental model with hypoxic exposure was initiated with 10 patients with OHT, 16 patients with NTG, and 18 controls. Regarding one OHT patient and four controls, the experimental setup was interrupted due to illness caused by altitude sickness. Hence, plasma samples were withdrawn from a total of 9 OHT patients, 16 NTG patients, and 14 controls, as outlined in the flow chart of the recruitment process in Figure A1.

## Appendix B

Plasma samples taken at the three time points throughout the hypoxia setup show the regulation of the analyzed markers for antioxidant capacity and oxidative stress in patients with OHT, NTG and controls. None of the analyzed markers were significantly regulated in response to hypoxic exposure (Figure A2, Figure A3, Figure A4 and Figure A5).
